# Epidemiology of Hepatitis C Virus Infection in Highly Endemic HBV Areas in China

**DOI:** 10.1371/journal.pone.0054815

**Published:** 2013-01-25

**Authors:** Duan Li, Yong Long, Tingcai Wang, Dan Xiao, Jingxia Zhang, Zhiwen Guo, Bo Wang, Yongping Yan

**Affiliations:** 1 Department of Epidemiology, and the Ministry of Education Key Lab of Hazard Assessment and Control in Special Operational Environment, School of Public Health, Fourth Military Medical University, Xi’an, China; 2 Wuwei Municipal Center for Disease Control and Prevention, Gansu, China; University of Cincinnati College of Medicine, United States of America

## Abstract

**Background:**

Wuwei City has the highest prevalence of hepatitis B virus (HBV) in China. From 2007 to 2011, the average reported incidence rate of hepatitis B was 634.56/100,000 people. However, studies assessing the epidemic features and risk factors of HCV in the general population of Wuwei City are limited.

**Methods:**

A total of 7189 people were interviewed and screened for HCV antibodies. HCV RNA and HCV genotypes were analyzed by PCR. Relevant information was obtained from the general population using a standardized questionnaire, and association and logistic regression analyses were conducted.

**Results:**

The anti-HCV prevalence was 1.64% (118/7189), and HCV-RNA was detected in 37.29% (44/118) of the anti-HCV positive samples. The current HCV infection rate was 0.61% (44/7189) in the Wuwei general population. Hepatitis C infection rate was generally higher in the plains regions (χ^2^ = 27.54,P<0.05), and the most predominant HCV genotypes were 2a (59.1%) and 1b (34.1%). The concurrent HCV and HBV infection rate was 1.37%, and a history of blood transfusion (OR = 17.9, 95% CI: 6.1 to 52.6, p<0.001) was an independent risk factor for HCV positivity.

**Conclusions:**

Although Wuwei is a highly endemic area for HBV, the anti-HCV positive rate in the general population is low. More than one-third of HCV-infected people were unaware of their infection; this may become an important risk factor for hepatitis C prevalence in the general population. Maintaining blood safety is important in order to help reduce the burden of HCV infection in developing regions of China.

## Introduction

Hepatitis C virus (HCV) infection is a major health problem worldwide. Currently, 130 to 170 million people worldwide are infected with HCV, and the annual increase is approximately 3.5 million [1]. It is estimated that about 0.2 to 26% of the general population in different countries are chronically infected by HCV [2]–[4].

In China, approximately 40 million people are infected with HCV [5], and it is estimated that 50% to 85% of all individuals infected with HCV develop chronic hepatitis; of these patients, 20% to 30% progress to liver cirrhosis that may lead to hepatocellular carcinoma (HCC). There is considerable geographic and temporal variation in the incidence and prevalence of HCV infection in China, and the prevalence of antibodies to HCV (anti-HCV) has been reported to vary considerably, ranging from 1.0% to 3.2% in most areas [6], [7].

HCV transmission is associated with sexual contact, injecting drug use, Tattooing and body piercing, etc. It is well known that HBV and HCV infections share modes of transmission; they are usually transmitted by parenteral exposure to infected blood products or via mother-to-child transmission [8]. Hepatitis B virus (HBV) infection is highly prevalent in China [9]. Wuwei city is one of the most under-developed areas in northwestern China. From 2007 to 2011, the average reported incidence rate of hepatitis B was 634.56/100,000 people, which is higher than the national average level (88.82/100,000 people) [10].

However, little is known about the HCV infection among the general population in this area, and there have been no reports on the potential relationship between HCV infection and the high prevalence of HBV infection. In order to assess the epidemic features of HCV prevalence in Wuwei, we conducted an epidemiological survey among the general population.

## Materials and Methods

### Sampling Method

The Proportional to Population Size (PPS) Cluster Sampling method was used in this study. The total natural population number in the Wuwei area was nearly two million, and 85% of the people are part of the rural Wuwei population. There are four administrative divisions: Liangzhou District, Gulang County, Minqin County, and Tianzhu Autonomic County, and each division consists of urban and rural populations. The total natural population number in the Wuwei area was divided into eight strata according to the four administrative divisions characterized by either urban or rural populations. The proportions of the populations in the eight strata were 11%, 41%, 1.3%, 20%, 3.5%, 12.3%, 2.3% and 8.6%, respectively, and these proportions were used as sample weights.

In rural areas, the sampling units were the villages and in urban areas, the sampling units were the communities. Each sampling unit weight was calculated by dividing the total number of the population in each stratum by the number in the sampling unit. The analysis was performed using Statistical Product and Service Solutions (SPSS version 15.0).

### Data Collected

In August 2011, trained social workers conducted an interview using a complete and detailed questionnaire to assess potential risk factors of HCV infection; the questionnaire included questions such as educational level, history of liver disease, family medical history, blood donation/transfusion history, blood transfusion, past use of glass syringes, and surgical intervention.

We used information from anti-HCV-positive people as the case group, and the control group was selected from people who were administered the questionnaire, but who were HCV-negative; people in the control group were age- and sex-matched HCV-negative people from the same region as those in the case group who were HCV-positive. This case-control study assessed the risk factors for HCV infection.

### Detection of Anti-HCV

A 3 ml serum sample from each person was collected, separated, labeled, and stored frozen at −20°C within 4 h of the collection. All the samples were screened for anti-HCV antibodies using commercial third generation enzyme immunoassays (ELISA) from two manufacturers (Wantai Core Anti-HCV EIA, C20110102; Kehua Core Anti-HCV EIA, C2101012061, China). Serum that reacted with both of the commercial kits were considered to be positive, and positive findings were considered to be an indicator of previous HCV contact.

### Nucleic Acid Extraction and Detection

The viremia of anti-HCV-positive people was evaluated by detecting HCV RNA in their blood. HCV RNA was isolated from the serum using the guanidinium isothiocyanate (GITC)–acid–phenol method (Chomeczynski and Saachi, 1987). The 5′untranslated terminal region (5′UTR) region was used for detection of HCV by a reverse-transcriptase polymerase chain reaction (RT-PCR) using the Amplicor HCV kit (Daangene, China). Internal reaction controls were used to evaluate technical or other problems encountered. The criteria for performing and interpreting the results of all the assays were as outlined in the manufacturers’ instructions.

### HCV Genotyping by Nucleotide Sequencing

Samples positive for HCV RNA were genotyped. The PCR product was subjected to nucleotide sequencing, to identify the HCV genotype. The sequencing reactions used 5 pmol of forward/reverse primers in a 50 ml volume (Amplicor HCV Kit, China, The lower limit is 10^3^ copy/ml). Brieﬂy, a 382 nt segment of the NS5B region was amplified using primers, NS5B 8278S (8258–8278, sense): 5′ TGATACCCGCTGYTTTGACTC3′ and NS5B 8618AS (8639–8618, antisense): 5′GTACCTGGTCATAGCCTCCGTG3′. The RT-PCR reaction was carried out under the following conditions: amplifications at 45°C for 10 min, 95°C for 15 min, followed by 40 cycles of amplification at 95°C for 15 sec, 58°C for 1 min, 72°C for 1 min, and final extension at 72°C for 7 min. PCR products were sequenced directly with the Prism Big Dye (Applied Biosystems, Foster City, CA) in an ABI 3100 DNA automated sequencer(Applied Biosystems, CA, USA). All sequences were analysed in both forward and reverse directions.

HCV genotype was determined after alignment with reference sequences from the HCV database (available at : http://hcv.lanl.gov/content/hcv-db/BASIC_BLAST/basic_blast.html) followed by phylogenetic analysis. Sequences were aligned using CLUSTAL_ X and then edited by BioEdit. Phylogenetic analysis was performed with MEGA 3.0. The neighbor-joining method was used with 1000 bootstrap replications (Kimura 2-parameter Substitution Model). The reference sequences used in phylogenetic analysis were obtained from the Genebank.

Our study was approved by the ethics committee of the Fourth Military Medical University and all participants signed informed consent forms before participating.

### Statistical Testing

Statistical tests were performed using the SAS 9.1 Software Package. The χ^2^ test or Fisher’s exact test was used to determine whether associations were statistically significant. The crude odds ratios (OR) and 95% confidence intervals (95% CI) were calculated to estimate if there were differences in potential risk factors between HCV-positive and HCV-negative people. Comparison among groups should involve multiple comparisons of multiple sample means. A two-sided p value below 0.05 was considered to be statistically significant.

## Results

### Detection of HCV Biomarkers

The populations in six villages and two communities were selected in Wuwei. There were 7189 people selected, of whom 85.4% were from the rural population. There were 3105 males and 4084 females, in the age range of 1 to 100 years, with a mean age 36.6 years and median age 38.5 years (Table 1).

**Table 1:** General features of 7189 people in Wuwei.

General data	Total (n = 7189)
Sex (F/M)%	4084/3105 (56.8/43.2)
Age (Mean±SD (range))	37.6±10 (1–77)
ALT (Mean±SD (range))	25.6±15 (6–698)
AST (Mean±SD (range))	27.1±13 (12–402)
HBsAg positive (%)	510 (7.1)
Anti-HBs positive (%)	3522(49)
Anti-HBc positive (%)	3163(44)
Anti-HCV positive (%)	118 (1.6)
HCV RNA positive (%)	44 (0.6)

Note: Data is enlisted as n (%) unless stated.

Of the 7189 people screened in Wuwei, 118 were repeatedly anti-HCV positive by ELISA, and the anti-HCV prevalence was 1.64%.

HCV-RNA (RT-PCR) was detected in 37.29% (44/118) of the ELISA-positive samples, which equates to a current HCV infection rate of 0.61% (44/7189) among the general population in Wuwei.

### The General Characteristics of HCV Infectors

Of the 118 HCV-positive people, 36 (30.51%) were male and 82 (69.49%) were female, while 100 (84.75%) were married and 18 (15.25%) were unmarried.

There were HCV-infected people in every age group, but there were significant differences in the anti-HCV rate among the different age groups (χ^2^ = 17.42, *P*<0.05). People older than 40 years of age had a significantly greater frequency of anti-HCV (2.26%), and represented 70% of the HCV infected persons (Table 2).

**Table 2:** Anti-HCV positive rate of different gender and age groups.

Age groups	Male			Female			Total		
	No.	Positive case	Positive rate (%)	No.	Positive case	Positive rate (%)	No.	Positive case	Positive rate (%)
≥40	1558	22	1.41	1982	58	2.93	3540	80	2.26*
20–40	672	5	0.74	903	15	1.66	1575	20	1.27
≤20	875	9	1.03	1199	9	0.75	2074	18	0.87
Total	3105	36	1.16	4084	82	2.01**	7189	118	1.64

* Significantly different from ≤20 age group (χ^2^ = 14.77, *P*<0.05).

** Significantly different from Male group (χ^2^ = 7.86, *P*<0.05).

There were significant regional differences in the prevalence rate (anti-HCV and HCV RNA) of hepatitis C (χ^2^ = 27.54,*P*<0.05, χ^2^ = 20.27, *P*<0.05, respectively) (Table 3). The anti-HCV and HCV RNA positive rates were highest in Liangzhou District, and these rates were 2.49% and 1.04%, respectively. Tianzhu County had the lowest prevalence rates of positive anti-HCV and HCV RNA results, which were 0.65% and 0.18%, respectively.

**Table 3:** Regional distribution of anti-HCV and HCV RNA in Wuwei.

	No.	Anti-HCV		HCV RNA	
		Positive case	Positive rate (%)	Positive case	Positive rate (%)
Liangzhou District	3170	79	2.49	33	1.04
Minqin County	1552	15	0.97	4	0.26
Gulang County	1382	17	1.23	5	0.36
Tianzhu Autonomic County	1085	7	0.65**	2	0.18**
Total	7189	118	1.64*	44	0.61*

* Significant differences among different areas (*P*<0.05).

** Significant differences between Tianzhu Autonomic County and Liangzhou District (P<0.01).

### HCV Genotype Distribution

The HCV genotype was available in 44 HCV-RNA-positive people. HCV genotype 2a was detected in 26 people (59.1%) and genotype 1b in 15 people (34.1%), while in 3 (6.8%) it was not possible to determine the HCV genotype. The HCV genotype distribution was not statistically different between the four areas that had an increased prevalence of HCV Infection.

**Figure 1:**
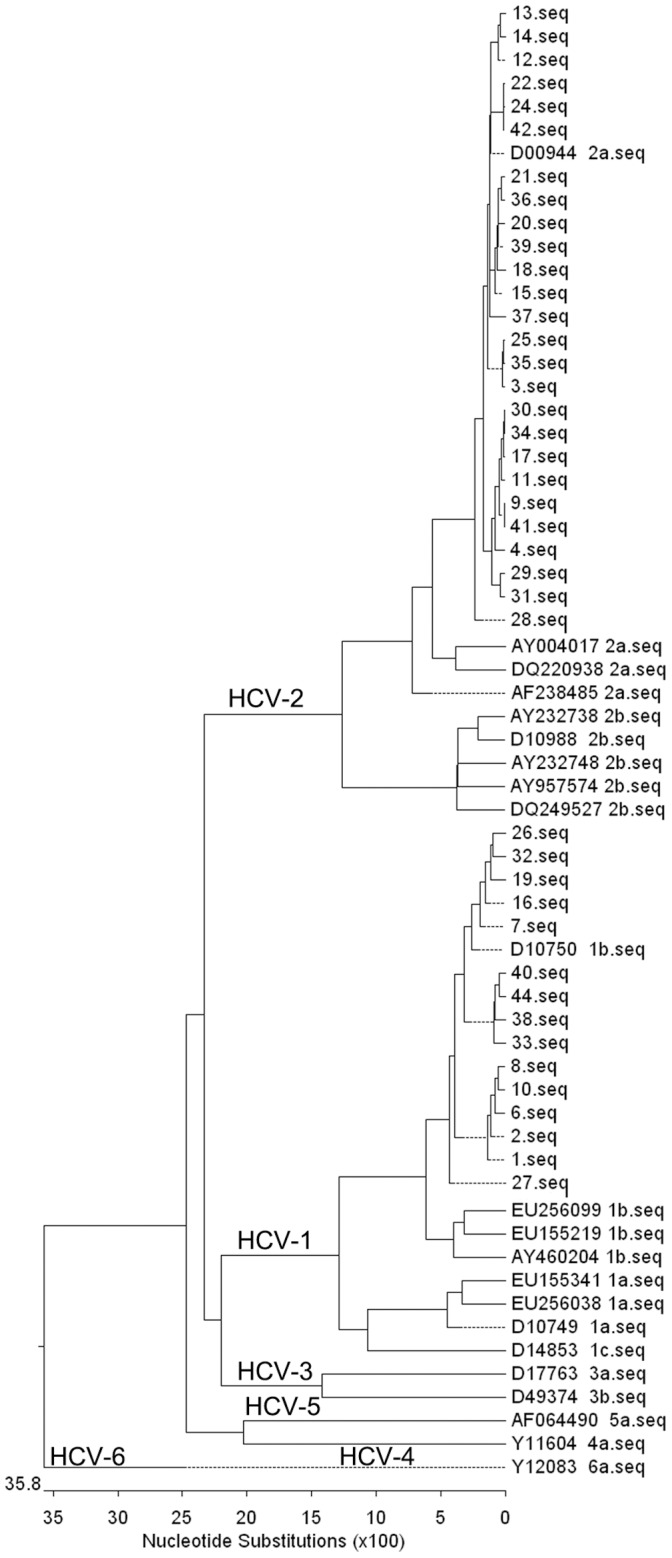
Phylogenetic tree. Phylogenetic tree of the NS5B region constructed with HCV-infected people isolates in Wuwei and reference sequences retrieved from international databases (GenBank).

All sequences generated in this study (the NS5B region nucleotide from 8278 to 8618) were subjected to phylogenetic analysis together with reference sequences of all major genotypes retrieved from the GenBank database. The majority of the HCV-infected people in Wuwei strains belonged to genotype 2a. Fig. 1 shows the phylogenies of the HCV strains obtained in this study along with closely related sequences and representatives of other genotypes.

### Concurrent HBV and HCV Infection

There were 510 people out of the population of 7189 in Wuwei City whose serum tested positive for HBsAg, and the HBV infection rate was 7.1%. All of the people infected with HBV had no antiviral treatment. There were 7 people who were anti-HCV-positive among the 510 HBsAg-positive patients. The HCV concurrent infection rate was 1.37%, slightly lower than the HCV infection rate (1.64%) of the local general population, but the difference was not significant (P>0.05).

### Risk Factors for HCV Infection

All 118 HCV-infected individuals and 236 HCV-negative persons were born in and/or were residents of Wuwei municipalities. The mean age (±SD) of HCV-positive and HCV-negative people was 36.6±13.2 years (range 2 to 76 years) and 37.1±12.8 years (range 2 to 75 years), respectively.

Table 4 shows the differences between the risk factors reported by HCV-positive and HCV-negative participants. Variables not significantly associated with HCV positivity included HBsAg and anti-HBc, blood donation, history of liver disease, gastroscopy and surgical intervention, and use of alcohol. Factors that were significant upon univariate analysis were included in a logistic regression model, and that remained statistically significant were a history of blood transfusion (P<0.0001, OR = 17.9, 95%CI = 6.1 to 52.6).

**Table 4:** Multivariate analysis of risk factors associated with HCV seropositivity among the general population in Wuwei.

Risk factors	anti-HCV positive people (N = 118) n (%)	anti-HCV negative people (N = 236) n (%)	OR (95% CI)	*P*
HBsAg positivity	7 (5.9)	22 (9.3)	0.75 (0.3–1.9)	0.538
Anti-HBc positivity	62 (52.5)	117 (49.6)	1.1 (0.7–1.8)	0.599
Blood transfusion	28 (23.7)	5 (2.1)	17.9(6.1–52.6)	<0.0001
Blood donation	5 (4.2)	8 (3.4)	0.8 (0.3–2.5)	0.690
History of liver disease	6 (5.1)	17 (7.2)	1.5 (0.6–3.8)	0.446
Gastroscopy	12 (10.2)	20 (8.5)	1.2 (0.6–2.6)	0.600
Surgical intervention	44 (37.3)	56 (23.7)	1.9 (1.2–3.1)	0.008
Use of alcohol^a^	13 (11.0)	20 (17.0)	1.3 (0.6–2.8)	0.438

Abbreviations: OR, odds ratio; CI, confidence interval; anti-HBc, antibody to hepatitis B.

Core; HBsAg, hepatitis B surface antigen; n = number of individuals in each group.

OR and *p* values were calculated by χ^2^ or Fischer’s exact test where applicable.

a Use of alcohol = frequent drinking (≥5 drinks per occasion or at least seven drinks per week).

In addition, among 118 HCV-infected individuals, no family clusters of HCV infection were found; only two families had more than one infected family member and other infected people were from different families.

## Discussion

Hepatitis C virus infection represents a worldwide health care problem. Once infected with HCV, only 20% of individuals can spontaneously remove the virus, and chronic hepatitis C may gradually progress to cirrhosis, hepatocellular carcinoma, and other serious liver diseases [11]. An epidemiology survey of hepatitis C infection is important for prevention and treatment of the disease. The HCV prevalence studies carried out in China during the past decades had limited geographical scope, different time frames, applied diverse methodologies, and predominantly focused on hospital based studies and high risk population groups including those who gave a blood donation or received a blood transfusion, drug abusers, individuals with HIV, patients receiving dialysis, and sex workers [12]–[14]. While there is information on HBV and HCV infections in the general population, there is still little information on HCV infection conditions in hepatitis B-prevalent areas.

The findings showed that although hepatitis B and hepatitis C have similar routes of transmission, and although there was no HCV vaccine for prevention of HCV, the HBV and HCV infection rates were significantly different in the general population. The anti-HCV-positive rate in the general population in Wuwei City was 1.64%, which was lower than the previously reported hepatitis C prevalence rate of 3.2% in the general population in China [15]. This result suggests that HCV transmission was lower in Wuwei. This study also found that the current infection HCV rate was only 0.61% in the general population in Wuwei, and that the alanine aminotransferase (ALT) levels were normal in 77.27% (34/44) of people currently infected with HCV. These patients were not being treated because of an unclear diagnosis, which caused subsequent HCV disease progression. Otherwise, the level of HCV RNA detection amongst individuals with HCV antibodies is lower than that reported in some other studies. We considered that Wuwei City was one of the most under-developed areas in northwestern China. Limited condition might cause some bias in the data collected.

There were significant regional differences in the hepatitis C prevalence rate. The Liangzhou District had the highest HCV prevalence rate (2.49%), which was 3.8 times of that in Tianzhu autonomic County, which had the lowest prevalence rate (0.65%). The hepatitis C infection rate was generally higher in the plains regions. Differences in HCV prevalence rates among the different areas indicated the need for extensive studies to determine the epidemiology of HCV infection and to develop appropriate prevention programs to control transmission of the virus.

Each age group contained an HCV infected person in Wuwei, but the anti-HCV positive rate increased with an increase in age, which is consistent with previous studies [16]. We hypothesize that with increasing age, there may be more opportunity for exposure to risk factors, resulting in increased infection rates. In addition, women had an anti-HCV-positive rate of 2.01%, which was higher than that of men (1.16%), and which may be a result of more chances for infection in women through procedures and situations such as curettage, abortion, childbirth, and other hematogenous routes. However, many men were not at home because they were working outside the home when their family members answered the study questionnaire, which could explain the relatively large number of female respondents in our study. This might cause some bias in the data collected, and the specific risk factors need further investigation.

In China, 1b is the predominant genotype [17], but there were significant differences among the regions. The rate of genotypes 2a gradually increases as you move from the south to the north in China. In this study, 93.18% (41/44) HCV-infected patients were infected with the HCV genotype 2a and 1b, but Genotype-2a was the most prevalent genotype in this studied population followed by genotypes 1b. This ruslt was different with the datas reported in the literature [18]. Probably because of the relatively small sample. More accurate HCV genotype distribution data in Wuwei need further investigation.

In general, the risk factors of HBV/HCV coinfection were found to be similar to those of HBV or HCV monoinfection. The survey found that there was HCV and HBV concurrent infection in the general population of Wuwei City, and among the HBsAg-positive patients, the HCV concurrent infection rate was 1.37%; this is slightly lower than the HCV infection rate (1.64%) of the local general population, which was significantly lower than that reported in the literature. It showed that there was a small amount of HBV and HCV coinfection, which may be because only 20% of respondents had received a blood transfusion, and thus the chance of becoming infected with HCV was greatly reduced.

Comparing HCV-positive and HCV-negative people, we found that the most prevalent risk factor in HCV-positive people in the general population was a history of blood transfusion (OR = 17.9). But 76.27% (90/118) of infected people had no definite risk factors for HCV infection. The survey also found that there were no family clusters of HCV infection, suggesting that HCV transmission within the family was not significant. However, over 30% of HCV-infected people in Wuwei did not know that they were infected; therefore, it may become an important risk factor for prevalence of HCV infection in the general population. Further investigation is needed to determine whether the transmission routes were related to the nature of the local peoples’ jobs, sexual contact, and other transmissions, or whether there were other routes of infection.

To our knowledge, this is the first study to highlight the epidemiology of HCV infection in HBV endemic areas in China. This hepatitis C epidemiological survey was conducted using a standardized questionnaire, a registration form, and various data processing forms, which included investigations and sensitive detection methods to ensure that the data collected was reflective of the HCV prevalence in the general population of Wuwei City.

Our findings are limited in scope due to the relatively small sample size, but they are directly relevant to the public health intervention strategies to prevent HCV transmission in Wuwei City, Gansu Province. Future research will involve detection of HCV in the general population to prevent missed diagnosis, misdiagnosis, and to prevent the HCV from becoming an epidemic.

In summary, our results provide an estimate of the prevalence of HCV infection in the general population of HBV endemic areas in China.
